# A New Species from the Canary Islands Increases the Diversity of the Red Algal Genus *Pterocladiella* in the Northeastern Atlantic

**DOI:** 10.3390/plants12020416

**Published:** 2023-01-16

**Authors:** Nereida M. Rancel-Rodríguez, Julio Afonso-Carrillo, Ana Tronholm, Marta Sansón

**Affiliations:** 1 Departamento de Botánica, Ecología y Fisiología Vegetal, Facultad de Ciencias, Sección Biología, Universidad de La Laguna, Avenida Astrofísico Francisco Sánchez, 38206 La Laguna, Spain; 2 Department of Biological and Environmental Sciences, University of Gothenburg, P.O. Box 461, 405 30 Göteborg, Sweden; 3 Gothenburg Global Biodiversity Centre, P.O. Box 461, 405 30 Göteborg, Sweden

**Keywords:** *cox*1, Gelidiales, morphology, Pterocladiaceae, *Pterocladiella canariensis*, *rbc*L, turf-forming algae

## Abstract

Environmental and human factors are inducing a drastic decline in many marine algae in regions with a high floristic richness as in the Canary Islands. Simultaneously, undescribed algal species continue to be discovered, suggesting a probable loss in diversity, before being properly identified and catalogued. Turf-forming Gelidiales occur in marine littoral communities from tropical to warm temperate regions and are challenging to identify correctly because of their small size and simple morphology. In the present study, we combined morphological and molecular phylogenetics methods to study a turf-forming species of the genus *Pterocladiella* from the Canary Islands (NE Atlantic). Both *cox*1 and *rbc*L gene analyses revealed a novel species described here, *Pterocladiella canariensis* sp. nov. The new species has no single unique morphological feature, but it is different by a distinctive combination of attributes, namely, minute size less than 18 mm in height, ribbon-like erect axes, small polygonal cortical cells, cystocarp circular in outline with placental tissue attached to the floor, spermatangial sori with sterile margins with spermatangia simultaneously formed on both sides of the blade, and tetrasporangia arranged in V-shaped rows. Phylogenies inferred from *cox*1 and concatenated genes (*cox*1 + *rbc*L) suggest a link to only two *Pterocladiella* species endemic to South Africa and Madagascar; nevertheless, the *rbc*L gene establishes *P. canariensis* as the earliest divergent lineage of the genus.

## 1. Introduction

The red algal genus *Pterocladiella* Santelices and Hommersand (Gelidiales, Rhodophyta) is an economically important source of agar and agarose with multiple uses in food and laboratory technologies [1,2,3]. It was established by Santelices and Hommersand [4] to accommodate those species previously assigned to *Pterocladia* J. Agardh exhibiting, together with the usual vegetative characters (i.e., compressed to flattened erect axes, opposite or alternate pinnate branches, terete to slightly compressed prostrate stolons bearing peg-like haptera, and internal rhizoidal filaments in the medulla), the following reproductive features: (1) intercalary carpogonia directed toward both surfaces of the thallus, (2) nutritive filaments growing centripetally and forming a virtually solid cylinder around the central axis, and (3) cystocarps usually attached to one side of the cystocarp floor with chains of carposporangia on the remaining three sides. In addition to the type species, *Pterocladiella capillacea* (Gmelin) Santelices and Hommersand, three other species were assigned to the new genus supported by both morphological and molecular evidence [5,6]. Subsequent studies led to the transfer of a total of ten species initially assigned to *Pterocladia* within *Pterocladiella* [7,8,9], and uncovered an ensemble of new species based on molecular and morphological datasets, mainly among small taxa with relatively simple morphologies growing in dense turf-like communities [1,2,10,11,12,13,14,15,16,17]. Currently, a total of 25 species with warm-temperate to tropical distributions are accepted in *Pterocladiella* [18]. However, recent findings by Boo et al. [19], using DNA-based species delimitation methods, have uncovered a notable cryptic diversity in *Pterocladiella*, delimiting up to 43 species, of which 19 remain undescribed, including two species hitherto assigned to *Gelidiella* Feldmann and Hamel. 

*Pterocladiella capillacea* and *P. melanoidea* (Schousboe ex Bornet) Santelices and Hommersand have been the only two species of the genus identified in the Northeast Atlantic and the Mediterranean Sea up until now [3,18,20]. But according to Boo et al. [19] a third species must be considered since the subtidal *Gelidiella calcicola* Maggs and Guiry [21] and the intertidal *P. melanoidea* sensu Díaz-Tapia and Bárbara [22] from Atlantic Europe are conspecific and different from *P. melanoidea* [19]. Both *P. capillacea* and *P. melanoidea* have also been reported from the Canary Islands [23,24]. The relatively widely distributed *P. capillacea* is a common species in macroalgal assemblages from shallow sublittoral in the Canaries [25], whereas *P. melanoidea* has been reported only from a few localities as a eulittoral turf-like species growing in low-light habitats [26,27,28]. Subsequent studies carried out on a population from the south of Tenerife revealed plants that, although assigned to *P. melanoidea*, exhibited vegetative and reproductive morphological features previously unreported in this species [29,30]. Here we examine in detail the attributes of these plants showing morphological and molecular evidence that support the description of the new species *Pterocladiella canariensis* N.M. Rancel-Rodríguez, J. Afonso-Carrillo, A. Tronholm and M. Sansón.

## 2. Results

### 2.1. Phylogenetic Analyses

**Figure 1:**
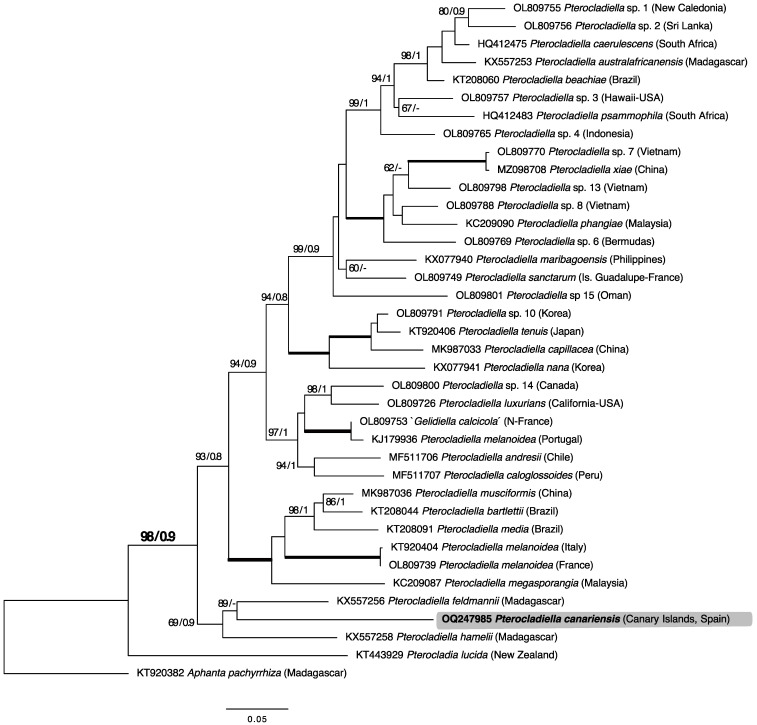
Maximum likelihood (ML) tree of forty-five (1397 bp) *rbc*L sequences calculated using the GTR+I+G evolution model. ML bootstrap values ≥ 60 and Bayesian inference posterior probability values ≥ 0.8 are shown for each branch. Black bold lines indicate full support in both analyses.

Two new sequences of *cox*1 and *rbc*L were obtained from a specimen from the Canary Islands. A total of 45 *rbc*L sequences (1397 base pairs) were aligned including publicly available sequences of *Pterocladiella* and four outgroups: *Aphanta pachyrrhiza* Tronchin and Freshwater, *Gelidium canariense* (Grunow) Seoane-Camba *ex* Haroun, Gil-Rodríguez, Díaz de Castro and Prud’homme van Reine, *Gelidium corneum* (Hudson) J.V.Lamouroux, and *Pterocladia lucida* (Turner) J. Agardh. The new sequences were assigned to the monophyletic *Pterocladiella* clade with high support in the *rbc*L tree (BS: 97; BPP: 0.9; Figure 1), but did not match any publicly available sequences and are here assigned to the new species *Pterocladiella canariensis*. The other NE Atlantic *Pterocladiella* species (*P. capillacea*, *P. melanoidea*, and ‘*Gelidiella calcicola*’) were resolved in distant topological positions from the Canarian species. The pairwise divergences were 0.165 for *P. canariensis* and *P. capillacea*, 0.200 with *P. melanoidea*, and 0.120 with ‘*Gelidiella calcicola*’.

**Figure 2:**
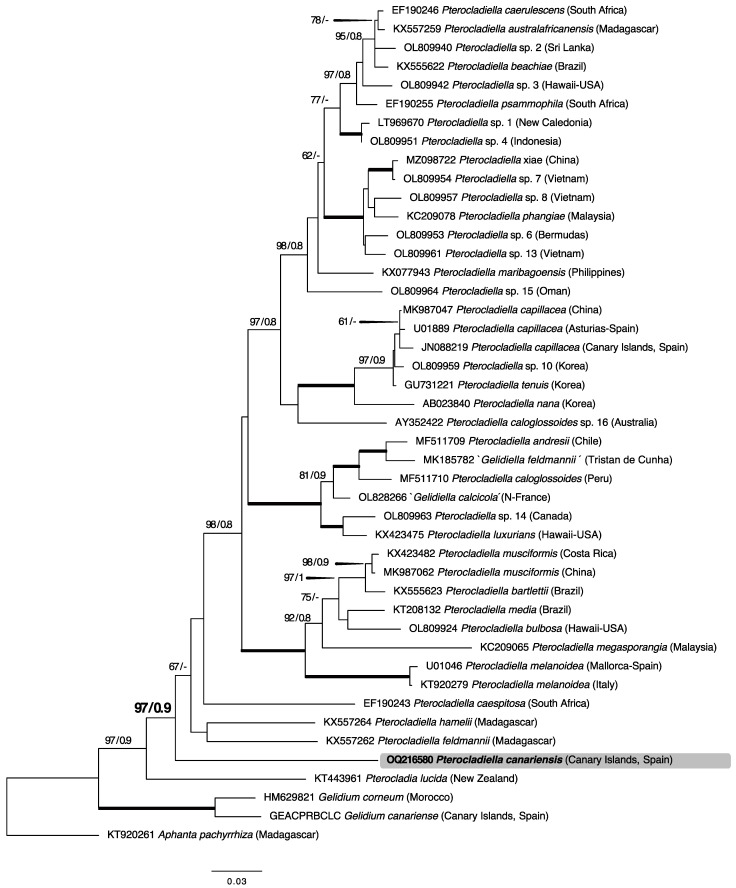
Maximum likelihood (ML) tree of 38 (1337 bp) *cox*1 sequences calculated using the GTR+I+G evolution model. ML bootstrap values ≥ 60 and Bayesian inference posterior probability values ≥ 0.8 are shown for each branch. Black bold lines indicate full support in both analyses.

Thirty-eight *cox*1 sequences (1337 bp) were aligned using two outgroups: *Aphanta pachyrrhiza* Tronchin and Freshwater, and *Pterocladia lucida* (Turner) J. Agardh. The new species is nested within the large clade of all remaining species of *Pterocladiella* with high support (BS: 98; BPP: 0.9; Figure 2). In the *cox*1 tree, *P. canariensis* clustered with *P. feldmannii* G.H.Boo, L.Le Gall, I.K.Hwang and S.M.Boo and *P. hamelii* G.H.Boo, L.Le Gall, I.K.Hwang and S.M.Boo (both from Madagascar) as a sister clade to the rest of the *Pterocladiella* species with low support (BS: 69; BPP: 0.9; Figure 2). The *cox*1 pairwise divergence between the Canarian specimens and *P. feldmanii* was 0.170.

**Figure 3:**
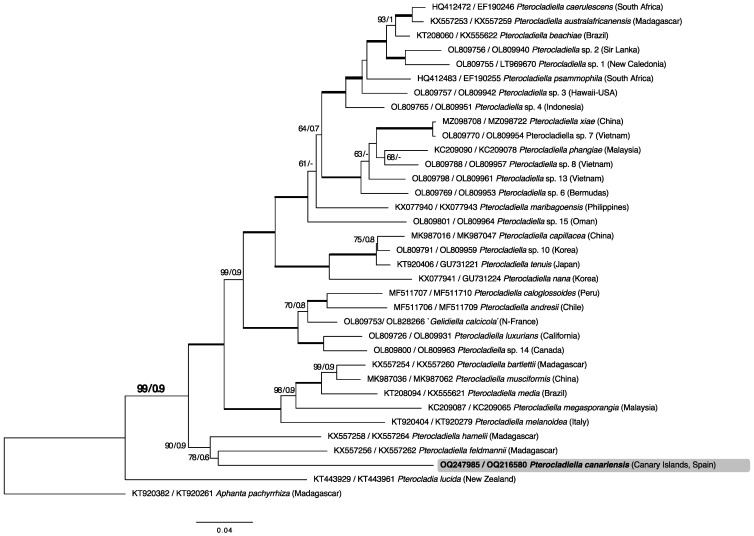
Maximum likelihood (ML) concatenated tree of 35 (2686 bp) *cox*1 and *rbc*L sequences calculated using the GTR+I+G evolution model. ML bootstrap values ≥ 60 and Bayesian inference posterior probability values ≥ 0.8 are shown for each branch. Black bold lines indicate full support in both analyses.

Both gene sequences from thirty-five taxa were concatenated and analyzed (2686 bp). The concatenated alignment was concordant with the *cox*1 gene phylogeny and the new specimen from the Canary Islands received strong support within the *Pterocladiella* genus (BS: 99; BPP: 0.9). *P. canariensis* is clustered with *P. feldmanii* and *P. hamelii* with better resolution than the *cox*1 gene (BP: 90; BPP: 0.9; Figure 3).

### 2.2. Morphological Analysis

#### 2.2.1. Habit and Vegetative Morphology 

**Figure 4:**
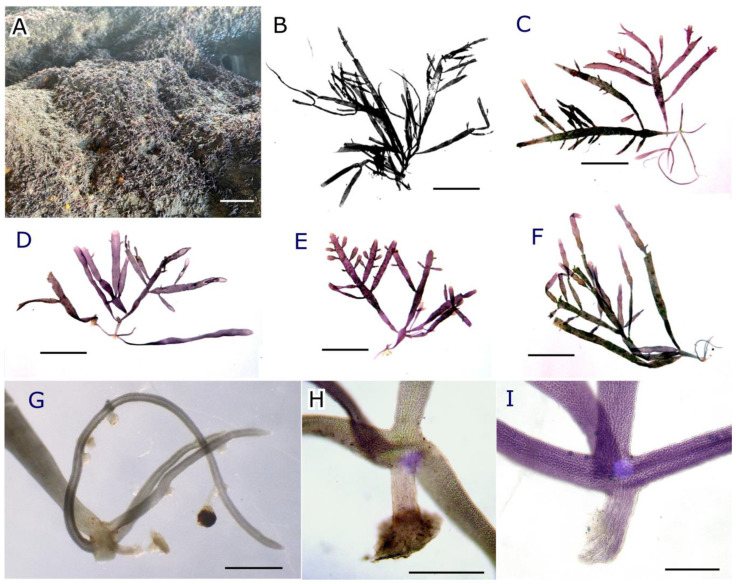
*Pterocladiella canariensis* sp. nov. (**A**) Habitat of *P. canariensis*, growing as turf-like tufts on rocky substrates; (**B**) holotype, preserved as a dry herbarium specimen (TFC Phyc 16400); (**C**–**F**) morphological variability of fresh specimens with branched erect axes arising from prostrate axes: (**C**) cystocarpic female gametophyte; (**D**) male gametophyte; (**E**,**F**) tetrasporophytes; (**G**) terete prostrate axes (stolon) with numerous holdfasts; (**H**) mature holdfast arising oppositely to the upright axis and ended by a small disc; (**I**) peg-like holdfast with acute end showing free rhizoidal filaments. Scale bar: 4 cm (**A**), 5 mm (**B**–**F**), 500 µm (**G**), and 300 µm (**H**,**I**).

Plants formed low-growing turf-like tufts that attached firmly to rocky substrates in low-light habitats (Figure 4A). Plants were dark red to deep purple when live and turned to a dark black color when dried as herbarium vouchers (Figure 4B–F), slender in appearance, membranous to slightly cartilaginous, consisting of stoloniferous and erect axes. The erect axes were flattened and ribbon-like, 300–600 µm wide and 40–70 µm thick, attenuated and subcylindrical towards the base, up to 30 µm wide. The main axes reach 6–11(–18) mm high, with distichous and very irregular, alternate, opposite, or pinnate branches. Branches were usually up two orders, attenuated at the base, irregular in width, and ending in acute or rounded tips (Figure 4B–F). Occasionally, lateral branches wholly or terminally filiform become prostrate and form secondary holdfasts. Sometimes erect axes are partially covered by dense populations of coccoid cyanobacteria. Prostrate axes (stolons) were terete to slightly compressed, 100–130 µm wide and 6–15(–22) mm long (Figure 4G), irregularly branched, fixed to the substrate by holdfasts usually arising oppositely to the upright axis (Figure 4H,I). Each holdfast consists of internal thick-walled rhizoidal filaments coalescing in a thick sheath, (95–)112–118(–226) µm long and (36–)44–58 µm wide, surrounded by pigmented cortical filaments. Younger attachments were peg-like with an acute or rounded end, while the more mature ones ended in a small disc (Figure 4H,I). Sometimes, the holdfasts lost the covering cortical filaments and the rhizoidal filaments became free.

**Figure 5:**
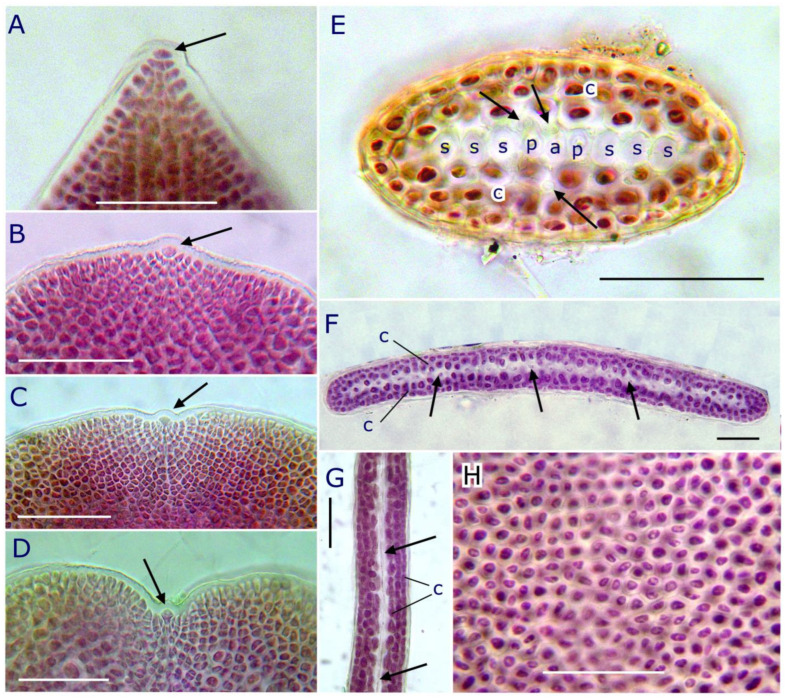
*Pterocladiella canariensis* sp. nov. (**A**) Attenuated apex with prominent apical cell (arrow); (**B**) obtuse apex showing a slightly protruding apical cell (arrow); (**C**) obtuse apex showing an apical cell (arrow); (**D**) apical cell (arrow) in a depression between cortical lobes; (**E**) transverse section of an erect axis through its attenuated and rounded basal portion showing the central row of non-pigmented thick-walled axial cell (a), periaxial cells (p), and second-order filament cells (s). Three to four layers of cortical cells (c) surround the central row. Some internal rhizoidal filaments (arrows) occur between the inner cortical cells; (**F**) transverse section of the distal flattened erect axis showing a central row of hyaline cells (arrows), and three layers of cortical cells (c); (**G**) longitudinal section of the flattened erect axis showing the axial filament (arrows), surrounded by the cortex; (**H**) surface view of an erect axis showing the irregular arrangement of the outer cortical cells. Scale bar: 50 µm.

Apical cell morphology varies from dome-shaped, 5–8 µm wide and 3–5 µm long, in attenuated apex, or slightly protruding in obtuse apices, or even sunken in a small depression between cortical lobes (Figure 5A–D). The first cell product of the apical cell divides to form an axial cell and two periaxial cells. In transverse-section, cells of the central row were rounded, 7–11(–13) µm wide, with a very narrow lumen, and included the narrower axial cell, and two opposite periaxial cells, from which arises a second-order filament of 3–5 cells (Figure 5E). Second-order filament cells form outwards short chains of up to three smaller globose, pigmented cortical cells (Figure 5E). Very few internal rhizoidal filaments, 3–4 µm wide, occurred irregularly arranged among the central row and the inner cortical cells, mainly in the erect axis (Figure 5E). This structural pattern, with a central row of hyaline cells and 3–4 layers of cortical cells, persists along the flattened erect axes (Figure 5F,G). In surface view, outer cortical cells were polygonal and isodiametric, thick-walled, 3–6 µm wide, and irregularly arranged (Figure 5H).

#### 2.2.2. Reproductive Morphology and Seasonality

Plants are dioecious and four morphologically similar plants can be recognized: sporophytes, male gametophytes, female gametophytes, and non-fertile plants. About 80% of the specimens collected exhibited reproductive structures throughout the year, except in winter when the fertile specimens were reduced to 50%. Sporophytes were always dominant among the fertile specimens with a ratio of 50%, which reduced to 30% in winter. Male gametophytes were in a low proportion throughout the year, but they represented up to 20% of the fertile specimens during the spring. Female gametophytes occur throughout the year, but they were especially abundant in autumn (20%).

**Figure 6:**
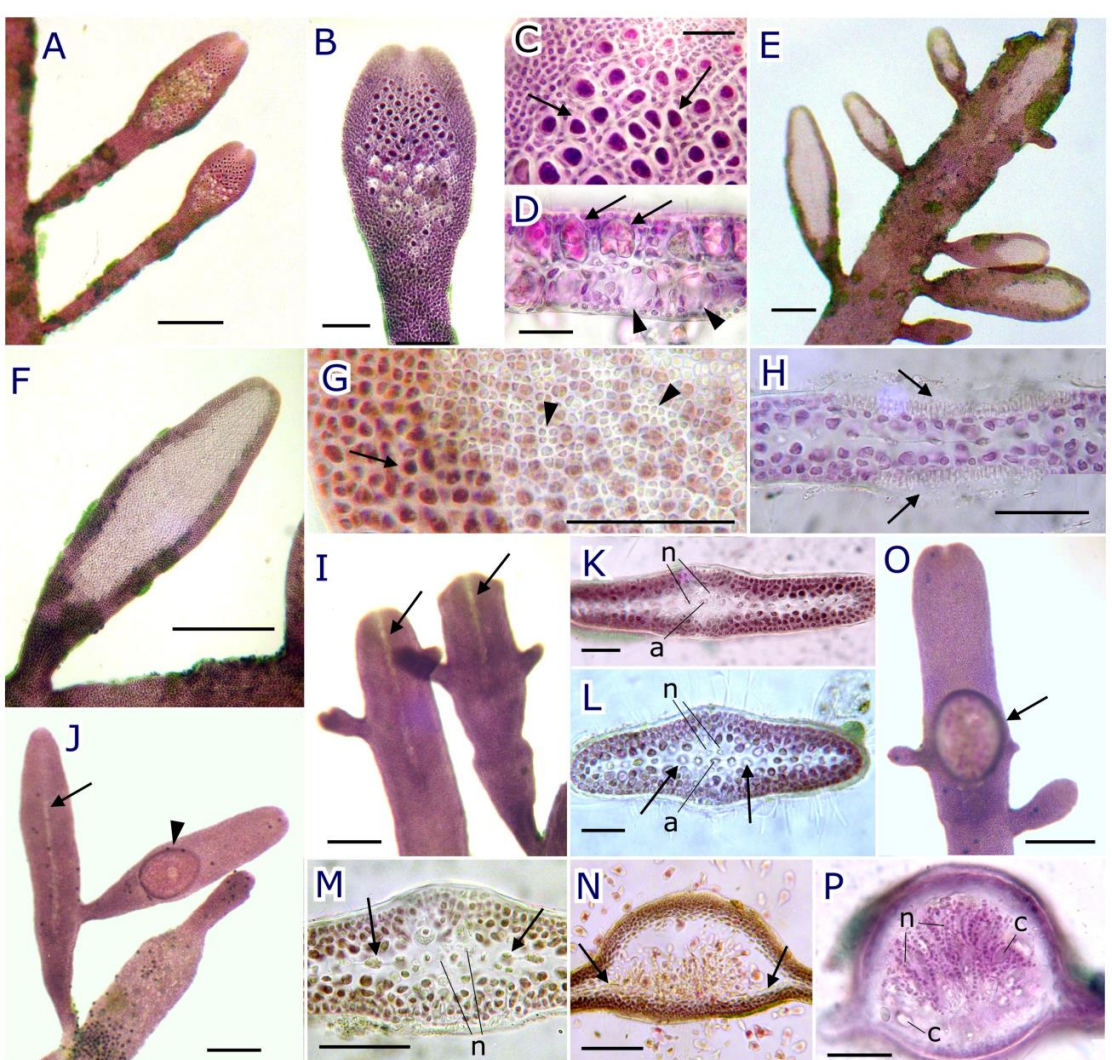
*Pterocladiella canariensis* sp. nov. (**A**) Two terminal tetrasporangial sori in lateral branchlets; (**B**) tetrasporangial sorus with sterile margin and tetrasporangia arranged in V-shaped rows; (**C**) surface view of rows of tetrasporangia (arrows) surrounded by outer cortical cells; (**D**) transverse section of an erect axis through a tetrasporangial sorus showing cruciate to decussately divided tetrasporangia (arrows) and empty cavities after tetrasporangia release (arrowheads); (**E**) spermatangial sori terminal in an erect axis and on shorth lateral branchlets; (**F**) spermatangial sorus with sterile margin; (**G**) surface view of spermatangial sorus showing pigmented outer cortical cells of the sterile margin (arrow) and densely packed hyaline spermatangia (arrowheads); (**H**) transverse section through a spermatangial sorus showing spermatangia arranged on both sides of the fertile branch (arrows); (**I**) two terminal fertile carpogonial axes showing longitudinal hyaline grooves starting from the apex (arrows); (**J**) branchlet with longitudinal hyaline groove (arrow) with lateral branch in which a cystocarp has developed (arrowhead); (**K**–**N**) successive phases of development of the cystocarp showing rounded nutritive cells (n) on both sides of the axial cell (a) and the central row (arrows) and the placental tissue attached to the cystocarp floor; (**O**) surface view of a mature cystocarp (arrow); (**P**) transverse section through a mature cystocarp showing the basal arrangement of placental tissue and the cavity occupied by nutritive filaments (n) and gonimoblastic filaments with terminal carposporangia (c). Scale bar: 300 µm (**A**,**E**,**F**,**I**,**J**,**O**), 100 µm (**B**,**N**,**P**), and 50 µm (**C**,**D**,**G**,**H**,**K**–**M**).

Tetrasporangia were formed in terminal sori on the flattened branches and branchlets (Figure 6A). Sori were elongated to ovoid, up to 780 µm long and 200–370 µm wide, with sterile margins (Figure 6B). Tetrasporangia were regularly arranged in V-shaped rows with 6–8 sporangia in each transverse row and developing in an acropetal sequence, with the most immature in the apical rows (Figure 6B,C). Occasionally, along the same branch or branchlets, two sori, initially isolated, can coalesce. Mature tetrasporangia were sub-spherical, often laterally compressed, 20–32 µm in diameter, usually cruciate or decussately divided, and immersed in the cortex (Figure 6D). Each tetrasporangium originated adaxially from an inner cortical cell, and in a transverse section tetrasporangia were arranged on both sides of the central row, increasing the thickness of the fertile branch to 70–105 µm. Mature sori showed discharged spores from the posterior rows, leaving gaps amongst the cortical cells (Figure 6B,D).

Spermatangia were formed in hyaline spermatangial sori on the terminal portions of axes or branchlets (Figure 6E). Sori were ovoid or elongated, 328–1165 µm long and 164–287 µm wide, showing a sterile margin (Figure 6F). Occasionally, two sori initially isolated coalesced. In surface view, hyaline spermatangia were globose, 1–3 µm in diameter, occupied the entire surface of the sorus, and simultaneously formed on both sides of the blade (Figure 6G). In transverse section, a mature spermatangial sorus appears slightly sunken and covered by a fine mucilaginous membrane that when broken allows the release of spermatia (Figure 6H). All outer cortical cells form closely packed, elongated spermatangial mother cells (c. 1 µm wide and 6–8 µm long), which cut off globose spermatangia outwards. Released spermatia can be observed adhering to the mucilage on the surface of mature sori.

Young fertile carpogonial branches are recognized by the presence of a longitudinal hyaline groove, 300–830 µm long, starting from the apex of the branch (Figure 6I,J). Carpogonia are formed from inner cortical cells in two parallel rows on each side of the axial filament but only towards one surface of the fertile branch. Nutritive filaments are borne on inner cortical cells on one or both sides around the axial cell, forming an incomplete ring around each axial segment (Figure 6K–N). A single subterminal cystocarp—rarely two to three—is formed on an axis or lateral branch. Mature cystocarps are ellipsoidal, 160–530 µm long, and 140–430 µm wide, with a sterile margin (Figure 6O). They are prominent, 350–540 µm high towards the fertile surface, and slightly convex on the opposite side. Cystocarps are unilocular, with one (two to three) ostioles irregularly placed, and 20–40 µm in diameter (Figure 6J,O). Ostioles are covered by a cuticle that detaches to release the carpospores. In transverse section, immature cystocarps exhibit a nutritive network of compact tissue around the central axis with rounded nutritive cells, 4–8 µm in diameter. Later, the gonimoblast attached to the cystocarp floor, and formed terminal chains of three to four ovoid carposporangia 12–21 µm long and 6–12 µm wide, that together with the nutritive filaments occupy most of a wide cystocarpic cavity that is circular in transverse-section (Figure 6P).

### 2.3. Pterocladiella canariensis N.M. Rancel-Rodríguez, J. Afonso-Carrillo, A. Tronholm and M. Sansón *sp. nov.*

Diagnosis: With morphological characters of the genus *Pterocladiella* and: attached to substrata by holdfast developing from prostrate axes (stolons); erect axes (up to 18 mm in height), compressed and ribbon-like (300–600 µm wide and 40–70 µm thick); sparse, irregular branching up to two orders; outer cortical cells polygonal (3–6 µm wide); very few internal rhizoidal filaments, restricted to inner cortical cells of basal portions of erect axes, and in prostrate axes; tetrasporangial sori with sterile margins and tetrasporangia arranged in V-shaped rows; spermatangial sori surrounded by a sterile margin with spermatangia simultaneously formed on both sides of the blade; cystocarps ellipsoidal (160–530 µm long and 140–430 µm wide) prominent and circular in outline in transverse section (350–540 µm high), with the placental tissue attached to the floor. 

Holotype: TFC Phyc 16400 (tetrasporophyte); El Médano, Tenerife, Canary Islands (28°02′44″ N; 16°32′14″ O), collected by M. Sansón on 22 September 2018 (Figure 4B). Isotypes: TFC Phyc 16401 (tetrasporophyte), TFC Phyc 16402 (tetrasporophyte), TFC Phyc 16403 (tetrasporophyte), TFC Phyc 16404 (female gametophytes), TFC Phyc 16405 (female gametophytes), TFC Phyc 16406 (female gametophytes), TFC Phyc 16407 (female gametophytes), TFC Phyc 16408 (male gametophytes), TFC Phyc 16409 (male gametophytes), and TFC Phyc 16410 (male gametophytes). 

Type locality: El Médano, Tenerife, Canary Islands.

Etymology: Specific epithet refers to the type locality (Latin: *canariensis*).

Molecular sequences of holotype specimen: OQ247985 for *cox*1 and OQ216580 for *rbc*L. Since the isotypes were duplicate specimens of the holotype, as indicated by the specimen number, DNA sequencing of the isotypes was not repeated.

Distribution: Only known from the type locality.

Other specimens studied: Information on other specimens examined in this study has been included in Materials and Methods.

## 3. Discussion

Integrative approaches combining morphological and molecular analyses involving the mitochondrial *cox*1 and plastid *rbc*L sequences have greatly improved species identification and phylogenetic relationships in the Gelidiales, as well as in other red algae [13,31,32,33,34,35]. For taxa such as *Pterocladiella* with poorly differentiated external appearance, relatively simple vegetative structure, high degree of phenotypic plasticity, and rare occurrence of sexual reproductive structures, establishing a sufficient number of morphological characters for the discrimination of species following the traditional taxonomy can be an unattainable goal without the help of molecular tools [2,17,36,37,38]. *Pterocladiella* represents a good example in this regard, taking into account the difficulty of studying small taxa growing in dense turf communities, which hide a variety of so far overlooked diversity [15,16,17]. Phylogenetic analyses using several molecular markers individually and in various combinations have proven to be very useful tools for establishing the differences and similarities among species not detected by morphological studies [1,2,14,39]. After the recent results presented by Boo et al. [19], *Pterocladiella* joins a growing list of red algal taxa such as *Lithophyllum* Philippi [40], *Portieria* Zanardini [41], *Polysiphonia* Greville [42], *Porolithon* Foslie [43], or *Sporolithon* Heydrich [44] for which DNA sequence analyses have resulted in a remarkable increase in species diversity. Pending morphological studies now underway [19], *Pterocladiella* currently includes 25 formally described species, to which should be added another 17 genetically differentiated but as yet undescribed species, and two species that must be transferred from *Gelidiella*.

The alga studied here and described as *Pterocladiella canariensis* N.M. Rancel-Rodríguez, J. Afonso-Carrillo, A. Tronholm, and M. Sansón sp. nov. grows in dense turf-like communities and it was initially assigned to *P. melanoidea*, the only species with similar characteristics previously reported for the NE Atlantic [29,30,45,46]. However, the morphological and molecular evidence presented here conclusively demonstrates that the specimens from the Canary Islands are distinct in vegetative and reproductive morphology and have a distant phylogenetic relationship with specimens identified as *P. melanoidea* in the Mediterranean Sea. 

*Pterocladiella canariensis* exhibits the characteristic vegetative and reproductive attributes of both Gelidiales and *Pterocladiella* [47,48]. Although it has no single species-specific feature, *P. canariensis* is distinguished from other species of the genus by a distinctive combination of attributes (Table 1). These include the minute size of plants that are less than 18 mm in height with erect axes compressed and ribbon-like, only subcylindrical in basal portions, sparse and irregularly branching up to two orders, small polygonal cortical cells (less than 6 µm wide), very few internal rhizoidal filaments that are restricted to the inner cortical cells of stolons and basal portions of erect axes, cystocarps circular in outline seen in transverse sections with the placental tissue attached to the floor, spermatangial sori surrounded by a sterile margin with spermatangia simultaneously formed on both sides of the blade, and tetrasporangia arranged in V-shaped rows in sori possessing a sterile margin (Table 1). However, *P. melanoidea* (type locality: Tangier, Morocco, and also reported in the Mediterranean Sea and Northeast Atlantic) is distinguished by larger plants (up to 40 mm in height) with thin erect axes subcylindrical to flattened, irregularly branched including opposite at right angles or pinnate branches up to two orders, larger ovoid cortical cells (6–12 µm long), and cystocarp of triangular outline seen in transverse section [45,46,47,49]. *Pterocladiella canariensis* is also distinct from ‘*Gelidiella calcicola’* Maggs and Guiry [21] (type locality: Carraroe, County Galway, Ireland), a morphologically similar taxon that has been erroneously identified as *P. melanoidea* in different localities of the NE Atlantic between Brittany and Portugal [22,50]. According to Boo et al. [19], this taxon belongs to *Pterocladiella* but it is phylogenetically distantly related to specimens identified as *P. melanoidea* in the Mediterranean Sea. ‘*Gelidiella calcicola*’ is characterized by plants up to 30 mm in height with terete to flattened erect axes, larger polygonal cortical cells (5–18 µm wide), triangular cystocarp in transverse section, and lacks sterile margin in tetrasporangial sori [21,22,50,51].

As compared in Table 1, *Pterocladiella canariensis* is also distinct from *P. australafricanensis*, *P. bartlettii*, *P. beachiae*, *P. capillacea*, *P. media*, *P. sanctarum*, and *‘Gelidiella feldmannii’*, the remaining species known from the Atlantic coasts. *P. australafricanensis* (type locality: Four Buoy Reef, Sodwana Bay, KwaZulu-Natal, South Africa; also reported from Brazil) is distinguished by the pinnate branching, the elliptical outline of cystocarp with placental tissue in the center of the cavity, and an irregular arrangement of tetrasporangia in sori lacking a sterile margin [13,39]. *P. bartlettii* (type locality: Saint Louis du Sud, Haiti; widely reported from Texas to Brazil, Madagascar, Malaysia, New Caledonia, Singapore, and Vietnam) differs by larger plants (up to 80 mm) profusely branched up to four orders, cylindrical to compressed erect axes, larger circular outer cortical cells, elliptical outline of the cystocarp, and lack of sterile margin in tetrasporangial sorus [2,39]. *P. beachiae* (type locality: Cahuita, Limón, Costa Rica; also reported from Brazil) mainly differs by larger plants (up to 60 mm) pinnately branched up to three orders, the elliptical outline of cystocarp with placental tissue in the center of the cavity, and an irregular arrangement of tetrasporangia in sori lacking a sterile margin [10,39]. *P. capillacea* (type locality: Mediterranean Sea; widely reported from the Atlantic and Pacific Ocean) is a larger plant (up to 20 cm) pinnately branched up to four orders, with numerous internal rhizoidal filaments in the central medulla, triangular and slightly rostrate cystocarp with placental tissue in the center of the cavity, and has tetrasporangia irregularly arranged or in horizontal rows [3,11,52]. *P. media* (type locality: Neptune Place, La Jolla, CA, USA; also reported from Brazil and Easter Pacific) mainly differs by erect axes distally branched up to three orders, the elliptical outline of cystocarp where the placental tissue is central in the cavity, and tetrasporangial sori lacking a sterile margin [39,53]. *P. sanctarum* (type locality: Îles des Saintes, Guadeloupe, West Indies; also reported from North American and Caribbean Islands) is also a minute species (up to 20 mm height) but differs by erect axes thin and compressed, ending narrower and cylindrical larger square outer cortical cells, and tetrasporangia irregularly arranged or in horizontal rows [9,54]. Finally, *‘Gelidiella feldmannii’* (type locality: Nightingale Island and the Settlement, Tristan da Cunha) is a taxon belonging to *Pterocladiella* according to molecular evidence [19,55], although to date no new name for this species has been proposed to date. It is also a minute species (up to 15 mm height) but differs by erect axes subcylindrical to flattened, irregularly branched including opposite at right angles or pinnate branches up to two orders [55,56].

**Table 1:** Comparison of *Pterocladiella canariensis* sp. nov. and Atlantic and related species.

Species/ References	Type Locality/ Distribution	Plant Size (mm)/ Branching	Erect Axes Shapes	Cortical Cells: Shape and Size in SV (µm)	Internal Rhizoidal Filaments	Cystocarp: Shape (TS)/ Placental Tissue Position in Cystocarp Cavity	Spermatangia: Sorus Margin/Spermatangial Arrangement	Tetrasporangia: Sorus Margin/Tetrasporangial Arrangement
*Pterocladiella australafricanensis* Tronchin and Freshwater [12,13,39]	South Africa/ Western Indo-Pacific and Western Atlantic	≤30/alternate pinnate up to two orders	lanceolate to ligulate, compressed	circular to elliptical 9.5–17	many in the outer medulla	elliptical/ central	n.d.	absent/ irregular
*P. bartlettii* (W.R.Taylor) Santelices [39]	Haiti/ Western Atlantic, Central Indo-Pacific, and Western Indo-Pacific	≤80/opposite to irregular up to four orders	linear, narrow, compressed to cylindrical	circular 7–13	few in the central medulla	elliptical/ close to the floor	n.d.	absent/ V-shaped rows
*P. beachiae* Freshwater in Thomas and Freshwater [10,39]	Costa Rica/ Western Atlantic	≤60/pinnate to irregular up to three orders	ligulate, compressed	circular to elliptical 8–15	many in the central medulla	elliptical/ central	present/ one or both branch surfaces	absent/ irregular
*P. caespitosa* (Kylin) Santelices [57]	South Africa/ South Africa	≤30/ rare to irregularly alternate up to two orders	thin, slightly compressed to flattened	rounded up to 15	many in the central medulla	circular/ close to the floor	n.d.	present/ irregular to V-shaped rows
*P. canariensis* N.M. Rancel-Rodríguez, J. Afonso-Carrillo, A. Tronholm and M. Sansón [Present study]	Canary Islands/ Canary Islands	≤18/sparse and irregularly branched up to two orders	compressed and ribbon-like, attenuate and subcylindrical towards the base	polygonal 3–6	few in the inner cortex of basal portions	circular/ close to the floor	present/ both branch surfaces	present/ V-shaped rows
*P. capillacea* (S.G.Gmelin) Santelices and Hommersand [3,11,52]	Mediterranean Sea/ Pacific, Atlantic, and Mediterranean Sea	≤200/pinnate up to four orders	slightly compressed to flattened	circular 4.5–9	many in the central medulla	triangular and slightly rostrate/ central	present/ both branch surfaces	present/ irregular to horizontal rows
*P. feldmannii* G.H.Boo, L.Le Gall, I.K.Hwang and S.M.Boo [2]	Madagascar/ Madagascar	≤60/opposite to irregular up to two (three) orders	linear and terete to compressed	polygonal 5–10 × 10–13	many in inner cortical layers	n.d.	n.d.	absent/ irregular
*P. hamelii* G.H.Boo, L.Le Gall, I.K.Hwang and S.M.Boo [2]	Madagascar/ Madagascar	≤30/ rare and irregular	thin and compressed	ovoid to rounded 12–13 × 14.3–20	few in the medulla	n.d.	n.d.	present/ irregular to horizontal rows
*P. media* (E.Y.Dawson) G.H.Boo and K.A.Miller [38,39,49]	California/ Eastern Pacific and Western Atlantic	≤40/irregular and distal up to three orders	linear and flattened	elliptical 4–7 × 7.5–9.5	rare in the medulla of basal portion of erect axes	elliptical/ central	n.d.	absent/ irregular to V-shaped rows
*P. melanoidea* (Schousboe ex Bornet) Santelices and Hommersand [45,46,47,48,49]	Morocco/ North-eastern Atlantic and Mediterranean Sea	≤40/irregular, opposite at right angles to pinnate up to two orders	thin, subcylindrical to flattened	ovoid 5–8 × 6–12	few in the medulla of basal portions	triangular/ close to the floor	present/ both branch surfaces	present/ V-shaped rows
*P. sanctarum* (Feldman and Hamel) Santelices [9,54]	Guadeloupe, West Indies/ Western Atlantic	≤20/ few and irregularly branched up to two orders	thin and slightly compressed, ending narrower and cylindrical	square 10–12	few in the medulla of basal portions	n.d.	n.d.	present/ irregular to horizontal rows
‘*Gelidiella calcicola*’ Maggs and Guiry [21,22,51]	Ireland/ NE Atlantic	≤30/ few to densely branched up to two orders	thin, terete to flattened	polygonal 5–18	few in medulla of stolons	triangular/ close to the floor	n.d.	absent/ V-shaped rows
‘*Gelidiella feldmannii*’ Baardseth [55,56]	Tristan da Cunha/Tristan da Cunha	≤15/ irregular, opposite at right angles to pinnate up to two orders	thin, terete, or flattened	n.d. 5–12	few in the medulla	n.d.	n.d.	present/ V-shaped rows

n.d.: no data available.

Our newly generated sequences formed a distinct monophyletic lineage indicating a separate and undescribed entity. Phylogenetic trees showed a distant relationship of *Pterocladiella canariensis* with any other currently sequenced species or the undescribed species molecularly differentiated by Boo et al. [19]. The new species is placed outside the large clade of all remaining species of *Pterocladiella*; based on the *cox*1 and *rbc*L + *cox*1 analyses it appears to be only linked to *P. feldmannii* and *P. hamelii* from Madagascar [2], in a heterogeneous set of early diverging species. The morphological characteristics of these species are compared in Table 1. *P. caespitosa* (type locality: Isipingo Beach, near Durban, South Africa) differs by thin, slightly compressed to flattened unbranched erect axes, or irregularly branched up to two orders, internal rhizoidal filaments numerous in the central medulla, and bigger rounded outer cortical cells [57]. *P feldmannii* (type locality: Nord-Est du phare d’Evatra, Madagascar) differs by plants of terete axes up to 60 mm with opposite to irregular branches, bigger polygonal outer cortical cells, internal rhizoidal filaments congested in the medulla and tetrasporangial sori without sterile margins and irregular arrangement of tetrasporangia [2]. Finally, *P. hamelii* (type locality: Lavanono, Madagascar) differs by plants up to 30 mm in height, erect, thin and compressed axes, simple or irregularly branched, outer cortical cells bigger ovoid to rounded, and tetrasporangia irregularly arranged or in horizontal rows [2].

A recent multigene time-calibrated phylogeny and ancestral area reconstruction have indicated that *Pterocladiella* most likely originated during the Early Cretaceous in the Tethys Sea, and ancient Tethyan vicariance and long-distance dispersal have shaped current distribution patterns [19]. An analysis of the geographic distribution of *Pterocladiella* species, based on specimens for which sequence data are available, has also shown that more than 81% of the species can be considered endemic since they have very restricted distributions [19], with the highest species diversity found in the Central and Western Indo-Pacific, whereas in other regions such as the eastern Atlantic species diversity was lower [19]. The finding of *P. canariensis*, so far only known from the type locality, increases the *Pterocladiella* diversity in the eastern Atlantic and it is in agreement with the thesis of a high proportion of endemic elements in this genus. The group of early diverging species (*P. caespitosa*, *P. feldmannii,* and *P. hamelii*) all with very restricted distributions, to which *P. canariensis* is now added could represent Tethyan relicts, such as those suggested by Boo et al. [19].

In recent years, as a result of environmental and human factors, numerous studies are showing a drastic decline in some species of Canarian marine algae that are at risk of extinction [58,59,60,61]. Simultaneously, undescribed algal species continue to be discovered [62,63,64], proof that current knowledge of the marine diversity of the Canary Islands is still incomplete, and that it is possible that part of the diversity is being lost when it has not yet been properly identified and catalogued.

## 4. Materials and Methods

### 4.1. Taxon Sampling and Morphological Analyses

Epilithic specimens were collected growing on rocky substrates as dense turf-like communities in low-light habitats in the lower eulittoral at El Médano, Tenerife (Canary Islands). Specimens were preserved in 4% formalin seawater for morphological studies, and/or pressed as herbarium sheets, and desiccated in silica-gel for molecular analysis. Selected fragments and hand sections were stained with 1% aniline blue acidified with 1% HCl, mounted in 50% Karo (Best Foods, Englewood Cliffs, NJ, USA) corn syrup solution, and examined using a Leica EZ4 stereomicroscope (Germany) and Zeiss microscope (Germany). Voucher specimens and microscopic slides were deposited in the Herbarium of the University of La Laguna, Spain (TFC) as follows: El Médano (Tenerife, Canary Islands): coll. J. Reyes and M. Sansón 02 March 1991, sporophytes and female gametophytes (TFC Phyc 7334); coll. J. Reyes and M. Sansón 14 May 1991, sporophytes, male and female gametophytes (TFC Phyc 7661, 7662); coll. N. Rancel and M. Sansón 02 October 2007, sporophytes (TFC Phyc 14119), female gametophytes (TFC Phyc 14120) and male gametophytes (TFC Phyc 14121); coll. N. Rancel, A. Tronholm and J. Rojo 13 November 2007, sporophytes (TFC Phyc 14122), female gametophytes (TFC Phyc 14123) and male gametophytes (TFC Phyc 14124); coll. A. Tronholm and J. Rojo 14 December 2007, sporophytes (TFC Phyc 14125), female gametophytes (TFC Phyc 14126) and male gametophytes (TFC Phyc 14127); coll. J. Rojo and V. Ramón 15 April 2008, sporophytes (TFC Phyc 14128), female gametophytes (TFC Phyc 14129) and male gametophytes (TFC Phyc 14130); coll. J. Rojo and L. Porzio 25 May 2008, sporophytes (TFC Phyc 14190), female gametophytes (TFC Phyc 14191), and male gametophytes (TFC Phyc 14192). Herbarium abbreviations follow Thiers [65]. Photomicrographs were made with a Nikon Coolpix 4600 digital camera (Japan) attached to a Zeiss microscope. 

### 4.2. DNA Extraction, Sequencing, and Phylogenetic Analyses

Total genomic DNA was extracted using the DNeasy PowerPlant Pro Kit (Qiagen, Germany), and subsequently purified with the Wizard^®^ DNA Clean-Up System (Promega Inc., Madison, WI, USA). Amplification of the *rbc*L and *cox*1 genes was conducted following the PCR conditions and primers outlined in Freshwater and Rueness [66], and Geraldino et al. [67]. PCR products were purified using ExoSAP-IT™ PCR Product Cleanup Reagent (Applied Biosystems™ by Thermo Fisher Scientific, Waltham, MA, USA) and sent for sequencing to Eurofins Genomics LLC (Louisville, KY, USA). Sequences generated in this study were deposited in GenBank and are publicly available under the following accession numbers: OQ216580 (*rbc*L) and xxxxxxx (*cox*1). Taxon sampling consisted of the newly generated sequences and a selection of sequences publicly available from GenBank (see Supplementary Table S1). Sequences were aligned using SeaView 4.7 [68], and phylogenies of *rbc*L and *cox*1 datasets were reconstructed using maximum likelihood (ML) and Bayesian inference (BI). The best-fitting nucleotide substitution model was selected using jModelTest v. 2.1.10 [69], with Akaike Information Criteria (AIC). ML analyses were performed using RaxML v8.2.10 [70], set as follows: a rapid bootstrap analysis and search for the best-scoring ML tree in one single run with 1000 bootstrap replicates under GTR+G+I model. The BI analyses were performed for individual datasets with MrBayes v3.2.7 [71], using the Metropolis-coupled Markov Chain Monte Carlo (MC3) with the GTR+G+I model. For each matrix, 100 million generations of two independent runs were performed with four chains and sampling trees every one hundred generations. Twenty-five percent of saved trees were removed as burn-in using Tracer version 1.7 [72], and the remaining trees were used to infer Bayesian posterior probabilities (BPP). Both methods were implemented using the Cyber Infrastructure for Phylogenetic Research (CIPRES Science gateway web server) [73].

## Data Availability

Not applicable.

## Supplementary Materials

The following supporting information can be downloaded at: https://www.mdpi.com/article/10.3390/plants12020416/s1, Table S1: List of published sequences with accession numbers used in the molecular analyses [74].
