# Supplementary material for: A New Species from the Canary Islands Increases the Diversity of the Red Algal Genus Pterocladiella in the Northeastern Atlantic
Source: Plants (Basel). 2023 Jan 16;12(2):416. doi: 10.3390/plants12020416 (PMC9866342; doi:10.3390/plants12020416)
Supplement: Supplementary file 1 [file plants-12-00416-s001.zip › plants-2095211-supplementary.pdf]

Table S1. List of publicly available sequences used in this study with GenBank accession numbers for both genes. Bold indicates sequence generated in the present study.

Species	State	*rbcL*	*cox*1	Reference
Pterocladiella capillacea	China	MK987047	MK987033	[14]
Pterocladiella capillacea	Spain	U01889	*	[5]
Pterocladiella capillacea	Spain (Canary Islands)	JN088219	*	[74]
Pterocladiella beachiae	Brazil	KX555622	KT208060	[39]
Pterocladiella australafricanensis	Madagascar	KX557259	KX557253	[14]
Pterocladiella caerulescens	South Africa	EF190246	HQ412472	[12]
Pterocladiella caespitosa	South Africa	EF190243	*	[12]
Pterocladiella maribagoensis	Philippines	KX077943	KX077940	[14]
Pterocladiella caloglossoides	Australia	AY352422	*	[14]
Pterocladiella caloglossoides	Peru	MF511710	MF511707	[14]
Pterocladiella andresii	Chile	MF511709	MF511706	[14]
Pterocladiella bartlettii	Rio do Fogo Beach, Maxarangua	KX555623	KT208044	[39]
Pterocladiella musciformis	Costa Rica	KX423482	*	[14]
Pterocladiella musciformis	China	*	MK987036	[16]
Pterocladia lucida	New Zealand	KT443961	KT443929	[14]
Pterocladiella melanoidea	Spain	U01046	*	[5]
Pterocladiella melanoidea	Italy	KT920279	KT920404	[14]
Pterocladiella melanoidea	Portugal	*	KJ179936	[22]
Pterocladiella melanoidea	France	*	OL809739	[19]
Pterocladiella tenuis	Korea	GU731221	*	[14]
Pterocladiella tenuis	Japan	*	KT920406	[14]
Pterocladiella feldmannii	Madagascar	KX557262	KX557256	[14]
Pterocladiella hamelii	Madagascar	KX557264	KX557258	[14]
Pterocladiella media	Brazil	KT208132	KT208091	[14]
Pterocladiella megasporangia	Malaysia	KC209065	KC209087	[14]

Species	State	*rbcL*	*cox*1	Reference
Pterocladiella luxurians	USA	KX423475	OL809726	[14]; [19]
Pterocladiella musciformis	China	MK987062	MK987036	[14]
Pterocladiella nana	Korea	AB023840	KX077941	[14]
Pterocladiella phangiae	Malaysia	KC209078	KC209090	[14]
Pterocladiella psammophila	South Africa	EF190255	HQ412483	[14]
Aphanta pachyrrhiza	Madagascar	KT920261	KT920382	[14]
Gelidium corneum	Morocco	HM629821	*	[14]
Gelidium canariense	Spain	GEACPRBCLC	*	[5]
Pterocladiella sp. 2	Sri Lanka	OL809940	OL809756	[19]
Pterocladiella sp. 3	Hawaii (USA); Indonesia	OL809942	OL809757	[19]
Pterocladiella sp. 4	Indonesia	OL809951	OL809765	[19]
Pterocladiella sp. 1	New Caledonia	LT969670	OL809755	[19]
Pterocladiella sp. 7	Vietnam	OL809954	OL809770	[19]
Pterocladiella sp. 8	Vietnam	OL809957	OL809788	[19]
Pterocladiella sp. 13	Vietnam	OL809961	OL809798	[19]
Pterocladiella sp. 6	Bermudas	OL809953	OL809769	[19]
Pterocladiella sp. 15	Oman	OL809964	OL809801	[19]
Pterocladiella sp. 10	Korea	OL809959	OL809791	[19]
Pterocladiella sp. 14	Canada; USA	OL809963	OL809800	[19]
Pterocladiella bulbosa	Hawaii (USA)	OL809924	*	[19]
Gelidiella calcicola	N-France	OL828266	OL809753	[19]
Gelidiella feldmannii	Africa	MK185782	*	[55]
Pterocladiella xiae	China	MZ098722	MZ098708	[17]
***Pterocladiella canariensis* sp. nov.**	**Spain**	**OQ216580**	**OQ247985**	**This study**
